# Andrographolide Inhibits Inflammatory Cytokines Secretion in LPS-Stimulated RAW264.7 Cells through Suppression of NF-*κ*B/MAPK Signaling Pathway

**DOI:** 10.1155/2017/8248142

**Published:** 2017-06-05

**Authors:** Yu Li, Shengnan He, Jishun Tang, Nana Ding, Xiaoyan Chu, Lianping Cheng, Xuedong Ding, Ting Liang, Shibin Feng, Sajid Ur Rahman, Xichun Wang, Jinjie Wu

**Affiliations:** ^1^College of Animal Science and Technology, Anhui Agricultural University, 130 West Changjiang Road, Hefei 230036, China; ^2^Institute of Animal Husbandry and Veterinary Medicine, Anhui Academy of Agriculture Sciences, Nongkenan Road, Hefei 230031, China

## Abstract

Andrographolide, the main active component extracted from* Andrographis paniculata* (Burm.f.) Wall. ex Nees, exerts anti-inflammatory effects; however, the principal molecular mechanisms remain unclear. The objective of this study was to investigate the molecular mechanisms of Andrographolide in modifying lipopolysaccharide- (LPS-) induced signaling pathway in RAW264.7 cells. An in vitro model of inflammation was induced by LPS in mouse RAW264.7 cells in the presence of Andrographolide. The concentration and expression levels of proinflammatory cytokines were determined by an enzyme-linked immunosorbent assay (ELISA) and quantitative real-time polymerase chain reaction (qRT-PCR), respectively. The nuclear level of NF-*κ*B was measured by an electrophoretic mobility shift assay (EMSA). The expression levels of NF-*κ*B, p38, ERK, and JNK were determined by western blot. Andrographolide dose-dependently inhibited the release and mRNA expression of TNF-*α*, IL-6, and IL-1*β* in LPS-stimulated RAW264.7 cells. The nuclear level of p65 protein was decreased in Andrographolide treatment group. Western blot analysis showed that Andrographolide suppressed LPS-induced NF-*κ*B activation and the phosphorylation of IkBa, ERK1/2, JNK, and p38. These results suggest that Andrographolide exerts an anti-inflammatory effect by inhibiting the activation of NF-*κ*B/MAPK signaling pathway and the induction of proinflammatory cytokines.

## 1. Introduction

**Figure 1:**
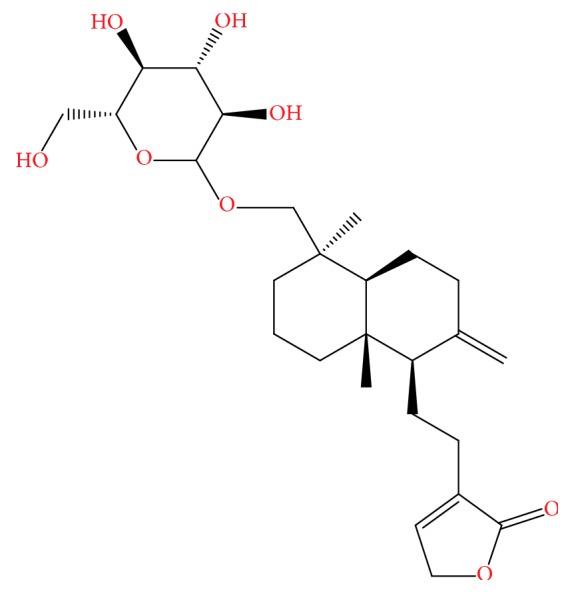
The chemical structure of Andrographolide.

Inflammation is a complex reaction that occurs within local or systemic animal organs in response to multiple endogenous or exogenous injuries, which mainly result in redness, swelling, fever, pain of organs, and tissue damage [1]. The* Andrographis paniculata* plant has been widely used for long periods in Asian traditional medicinal [2]. Common* Andrographis* herb is derived from* Andrographis paniculata* (Burm.f.) Wall. ex Nees. The whole plant or aerial parts of plant are employed for the purposes like cooling properties and a bitter taste that can reduce heat, detoxify, cool the blood, and reduce swellings [3]. Andrographolide (C_20_H_30_O_5_, Figure 1), a labdane diterpenoid that is produced by* Andrographis paniculata* plant, has been reported to have several pharmacological properties, including antibacterial, antiviral, and antiplatelet properties, stimulation of cell differentiation, protection of the liver, being a cholagogue, antitumor properties, and immunoregulation [4]. Previous studies reported that Andrographolide can inhibit the increase in capillary permeability caused by xylene and acetic acid in mice. Moreover, Andrographolide has exhibited obvious anti-inflammatory properties in response to the rat model of hind paw edema induced by egg white protein and carrageenan [5]. Andrographolide can inhibit the expression of inducible nitric oxide synthase (iNOS) by LPS-activated macrophages and the production of prostaglandin E2 [6]. However, the potential pharmacodynamic function and mechanisms by which Andrographolide exerts such anti-inflammatory properties remains unclear [7].

LPS is considered as one of the most potent inducers of proinflammatory factors [8], and bacterial infections often cause a strong inflammatory reaction. Macrophages are important immune activating cells and play a critical role in the induction of the inflammatory response in response to pathogen invasion [9]. Mouse macrophage RAW264.7 cells stimulated by LPS can produce inflammatory mediators and inflammatory factor, such as TNF-*α*, IL-1*β*, and IL-6 [10, 11]. Therefore, an excessive production of these proinflammatory cytokines can cause severe damage to surrounding tissues.

Studies have confirmed that LPS induces inflammation primarily by stimulating NF-*κ*B and the MAPK signaling pathway [12]. Therefore, inhibition of the inflammatory response by targeting signal transduction pathways has become a novel avenue for the treatment of inflammation.

In the present study, LPS was used to activate the NF-*κ*B and MAPK signaling pathways as the theoretical basis to investigate mechanisms of the anti-inflammatory effects of Andrographolide in LPS-induced inflammation of RAW264.7 cells. The aim of current investigation was to provide a theoretical basis for the further development of anti-inflammatory drugs.

## 2. Materials and Methods

### 2.1. Reagents

Andrographolide (Control of Pharmaceutical and Biological Product, Beijing, China), dimethyl sulfoxide (DMSO), fetal bovine serum, and 0.25% trypsin were purchased from HyClone (Logan, UT, USA); LPS (*Escherichia coli* 055:B5) was purchased from Sigma Chemical CO. (St. Louis, MO, USA); Mouse TNF-*α*, IL-1*β*, and IL-6 ELISA kits were purchased from Sbjbio company (Nanjing, China); primary antibodies of rabbit mAb p65, rabbit mAb ERK, and rabbit mAb JNK; secondary antibodies of goat anti-rabbit IgG antibody; and goat anti-mouse IgG antibody were purchased from Wuhan Boster Biological Engineering Co. (Wuhan, China); primary antibodies of rabbit mAb I*κ*B*α*, mouse mAb phospho-I*κ*B*α*, mouse mAb phospho-p65, mouse mAb phospho-p38, mouse mAb phospho-specific p42-p44 ERK, and mouse mAb phospho-specific p46-p54 JNK were purchased from Beyotime Biotechnology Co. (Nanjing, China).

### 2.2. Culture of RAW264.7 Cells

RAW264.7 mouse monocyte-macrophage cells were received as a gift from Jilin University (Changchun, China). RAW264.7 cells were inoculated in cell culture bottles, and then the cells were placed in an incubator at 37°C and 5% CO_2_. The culture medium (DMEM medium with 10% fetal bovine serum, 1% Gln, and 100 U/ml penicillin-streptomycin) were changed according to the cell growth conditions and color of the media, respectively. When the cells reached 80% confluence, the cells were subcultured by replacing the culture medium and the adherent cells were aspirated with a pipette, washed, and then seeded into new cell culture bottles to be incubated.

### 2.3. Microscopic Observation and Cell Counting Kit-8 (CCK-8) Assay for Cell Viability

Briefly, RAW264.7 cells in the logarithmic phase were plated at a density of 1 × 10^5^ cells/ml into 96-well plates in a 37°C, 5% CO_2_ incubator for 24 h. Subsequently, 100 *μ*L cells were added to each well, and the cells were treated with indicated concentrations of Andrographolide for 1 h in the incubator. When the cell morphology observed by microscope was adequate, the same concentration was being performed in triplicate, followed by stimulating with 1 *μ*g/ml LPS in a 37°C, 5% CO_2_ incubator for 18 h. A volume of 10 *μ*L CCK-8 (Dojindo, Kumamoto, Japan) was added to each well, and the cells were further incubated for additional 1–4 h. The optical density (OD) was measured at 450 nm using a Bio-Rad Model 550 Microplate Reader (Bio-Rad, Hercules, CA, USA), and the cell viability was calculated.

### 2.4. ELISA Assay for the Contents of Proinflammatory Cytokines

The RAW264.7 cells (2 × 10^6^ cells/ml, with 2 ml in each well) were seeded into six-well plates and incubated in the presence of different concentrations (6.25, 12.5, and 25 *μ*g/ml) of Andrographolide for 1 h, followed by stimulating with LPS (l *μ*g/ml) for 18 h [13]. The cell-free supernatants were subsequently employed to quantify the contents of proinflammatory cytokines (TNF-*α*, IL-1*β*, and IL-6) using a mouse ELISA kit, according to the manufacturer's instructions (Sbjbio company, Nanjing, China).

### 2.5. Total RNA Isolation and qRT-PCR

The RAW264.7 cells (2 × 10^6^ cells/ml, with 2 ml in each well) were seeded into six-well plates and incubated in the presence of different concentrations (6.25, 12.5, and 25 *μ*g/ml) of Andrographolide for 1 h, followed by stimulation with LPS (l *μ*g/ml) for 18 h. The supernatant was removed, and the sediments were washed with PBS twice. The total RNA was extracted using Trizol reagent following the manufacturer's instructions (TaKaRa, Dalian, China), and the RNA was reverse-transcribed into cDNA according to the Reverse Transcription System's instructions (TaKaRa, Dalian, China). According to the GenBank sequence, the primer sequence of the target genes (i.e., TNF-*α*, IL-1*β*, and IL-6) and the *β-actin* gene were designed using the software Primer Premier 5.0 (Table 1). The amplification products were analyzed by 1.5% agarose gel electrophoresis and a gel imaging and analysis system (UVItec, Cambridge, UK).

### 2.6. EMSA Assay for Protein Transportation to the Nucleus

RAW264.7 cells (2 × 10^6^ cells/ml, with 1 ml in each well) were seeded into six-well plates; 2 ml was added to each plate and incubated in the presence of different concentrations (6.25, 12.5, and 25 *μ*g/ml) of Andrographolide for 1 h when the cells reached 5 × 10^6^~1 × 10^7^ cells/ml. Following this incubation, the cells were stimulated with LPS (l *μ*g/ml) for 18 h; after that cells were collected and washed twice with ice-cold PBS, centrifuged at 500*g* and 4°C for 3 min, the supernatant was removed, and packed cell volume was estimated. The nucleoprotein was extracted with nuclear protein extraction reagent (Sagon, Shanghai, China) and the total protein was determined using the Bradford method (Biosharp, China). A 6.5% polyacrylamide gel was produced and made into a gel slab, an EMSA binding reaction was performed, and the samples were produced and dispensed.

Electrophoresis was initiated with a 0.5x TBE as the running buffer at 10 V/cm. When the bromophenol blue in the EMSA/Gel-Shift loading buffer ran to the lower edge of the gel, the electrophoresis was stopped. The required gel was cut, and the protein and probe (5′-AGTTGAGGGGACTTTCCCAGGC-3′) complexes were transferred into a nylon membrane (GE Healthcare, USA) with a transfer buffer at 254 nm, 120 mJ/cm^2^ in the cross-linking machine. After 45–60 s, cross-linking was completed, the sealing and washing liquid was dissolved at 37–50°C in a water bath, the nylon membrane was blocked with 15 ml sealing liquid for 15 min on a horizontal rotator, and the sealing liquid was removed. The new sealing liquid containing streptavidin-HRP conjugate (1 : 2000 dilution) was introduced; the membrane was shaken for 15 minutes on the horizontal rotator, followed by washing (4 × 5 min) with the washing liquid. The nylon membrane was then placed in a container with 20–25 ml of a determined equilibrium liquid and shook for 5 min. Then the nylon membrane was removed and the extra liquid was absorbed with absorbent paper. The chemiluminescent nucleic acid detection module working solution was added until the membrane was covered completely and then left at room temperature for 2-3 min. The nylon membrane was removed and the extra liquid was placed into the middle of two pieces of plastic wrap and tested using the G:BOX chemiXR5 Gel Imaging System. The results were analyzed using the Gel-Pro32 software (Syngene, Cambridge, UK).

### 2.7. Western Blot Analysis

RAW264.7 cells at 4 × 10^5^ cells/ml were seeded into six-well plates (2 ml per plate) and incubated for 24 h and then pretreated with different concentrations (6.25, 12.5, and 25 *μ*g/ml) of Andrographolide for 1 h. The cells were stimulated with LPS (1 *μ*g/ml) for 18 h and then collected and washed twice with ice-cold PBS. The total protein from the cells was extracted using a RIPA lysis buffer solution (Wuhan Boster Biological Engineering Co., Wuhan, China), and the total protein concentration was determined using a BCA Protein Assay Kit (Beyotime Inst. Biotech, Peking, China). The protein was split and the SDS-PAGE loading buffer (Beyotime Biotechnology Co., Shanghai, China) was added and placed into a 100°C water bath for 10 min and preserved at 4°C for later use.

The gel board was installed; a 12% separating gel and 15% stacking gel were produced; then five samples of proteins were added. Electrophoresis was initiated at 80 V and changed to 120 V after 25 min and continued until the samples run to the bottom of the gel. The required gel was cut and transferred into a PVDF membrane (Shanghai Jinsheng Biological Engineering Co) with electrophoresis buffer. The resulting membrane was blocked with 5% BSA for 4 h on a horizontal rotator at room temperature and then incubated with the primary antibodies at 4°C overnight. Subsequently, the membrane was washed with TBST three times followed by an incubation on the horizontal rotator for 5 min and incubated with the secondary antibody at room temperature for 45 min on the horizontal rotator. The blots were washed again with TBST three times for 5 min and then tested by the Gel Imaging System (Bio-Rad, Hercules, CA, USA). The results were analyzed using quantity one software (Bio-Rad, Hercules, CA, USA).

### 2.8. Statistical Analysis

Date were presented as mean ± SEM. Differences between the mean values of the normally distributed data were analyzed using one-way ANOVA (Dunnett's *t*-test) and a two-tailed Student's *t-*test. The criterion for the differences was considered significant at *P* < 0.05 or *P* < 0.01 in all studies.

## 3. Results

### 3.1. Effect of Andrographolide on Cell Viability

**Figure 2:**
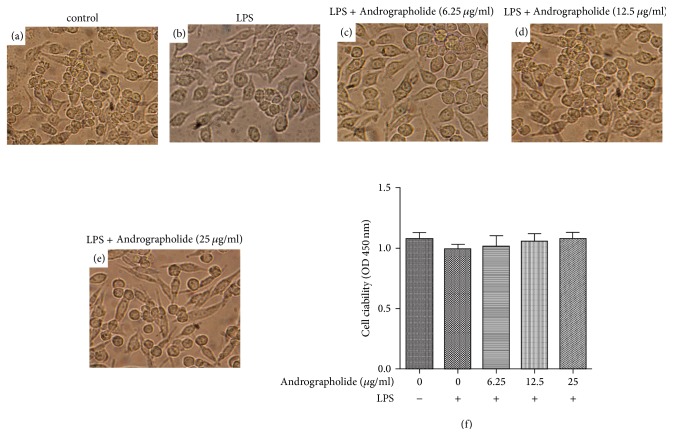
Effect of Andrographolide on morphologic changes and viability of RAW264.7 cells. (a) represents cells in control group; (b) represents cells treated by LPS; (c)–(e) represent cells treated with different concentration Andrographolide (6.25, 12.5, and 25 *μ*g/ml) for 1 h and LPS for another 18 h, respectively; (f) represents the effect of Andrographolide on the cell viability of RAW264.7 cells in control, LPS, and Andrographolide treated groups. Results are representative of three (f) independent experiments.

To evaluate the effects of Andrographolide (6.25, 12.5, and 25 g/ml) on RAW264.7 cells morphologic changes and viability, we performed inverted light microscopy (Nikon, Tokyo, Japan) and CCK-8 assay. As shown in Figures 2(a)–2(f), the cell morphology and viability of RAW264.7 cells have no recognizable changes with the increasing of Andrographolide concentrations. Cell viability in experimental groups with different doses of Andrographolide did not show any significant difference compared with the control group. These results indicated that the Andrographolide used in this study has no toxic effect.

### 3.2. Effect of Andrographolide on the Secretion of Proinflammatory Cytokines in LPS-Stimulated RAW264.7 Cells

**Figure 3:**
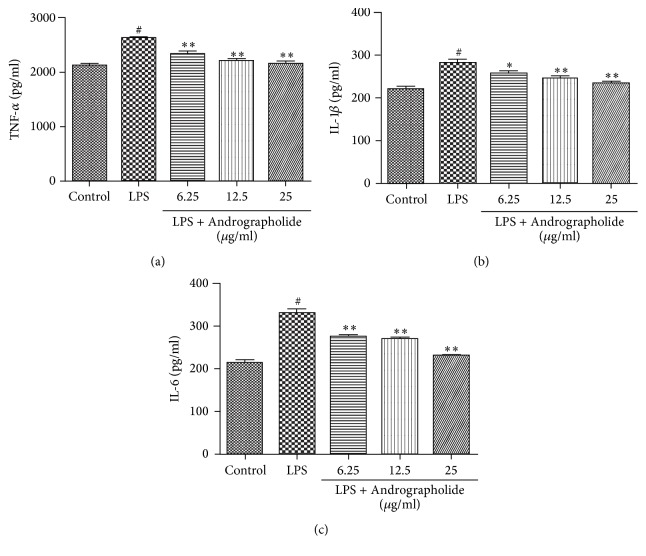
Effect of Andrographolide on the secretion of TNF-*α*, IL-1*β*, and IL-6 induced by LPS. # indicates the control group compared with the LPS group (*P* < 0.05); *∗* and *∗∗* indicate the Andrographolide group compared with the LPS group (*P* < 0.05) and (*P* < 0.01). Results of TNF*α* (a), IL-1*β* (b), and IL-6 (c) secretion are representative of three (a–c) independent experiments.

To evaluate the effects of Andrographolide on the secretion of the proinflammatory cytokines in LPS-stimulated RAW264.7 cells, we measured the level of each cytokine using an ELISA. As presented in Figures 3(a)–3(c), the levels of proinflammatory cytokine in the LPS group were significantly higher than that in the control group (*P* < 0.05). Moreover, the proinflammatory cytokine levels of the Andrographolide groups (doses of 6.25, 12.5, and 25 g/ml) were significantly lower than that in the LPS group (*P* < 0.05 or *P* < 0.01) and gradually decreased with the increasing doses of Andrographolide.

### 3.3. Effect of Andrographolide on the Expression Level of the Proinflammatory Cytokines in LPS-Stimulated RAW264.7 Cells

**Figure 4:**
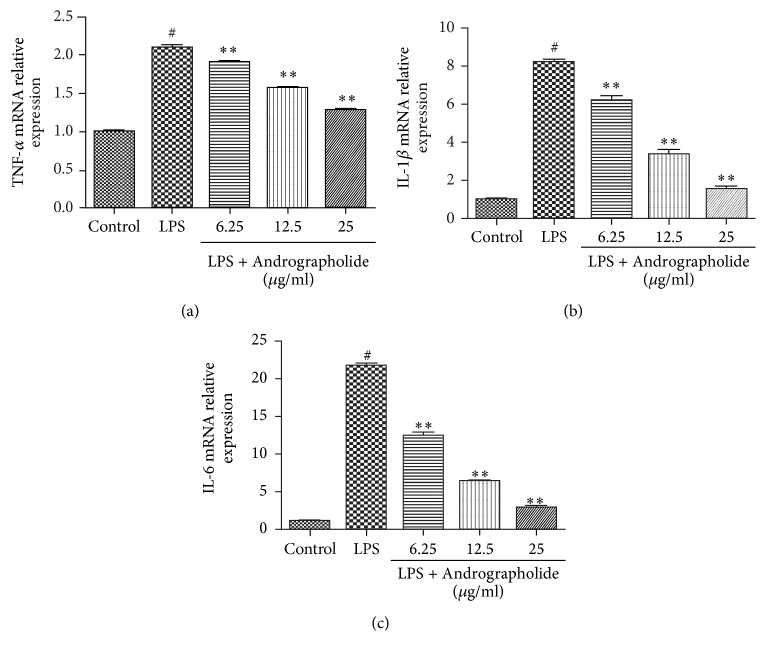
Effect of Andrographolide on the mRNA expression of TNF-*α*, IL-1*β*, and IL-6 induced by LPS. # indicates the control group compared with the LPS group (*P* < 0.05) and *∗∗* indicates the Andrographolide group compared with the LPS group (*P* < 0.01). Results of TNF*α* (a), IL-1*β* (b), and IL-6 (c) mRNA expression are representative of six (a–c) independent experiments.

Next, we sought to evaluate the effects of Andrographolide on the expression level of the proinflammatory cytokines in LPS-stimulated RAW264.7 cells by qRT-PCR. As shown in Figures 4(a)–4(c), the mRNA expression levels of the proinflammatory cytokines in the LPS group were significantly higher than that in the control group (*P* < 0.05). Moreover, the mRNA expression levels of the proinflammatory cytokines in the Andrographolide groups (doses of 6.25, 12.5, and 25 g/ml) were significantly lower than that in the LPS group (*P* < 0.01) and decreased in a dose-dependent manner.

### 3.4. Effect of Andrographolide on the Nuclear Level of NF-kB Probe Binding Activity

**Figure 5:**
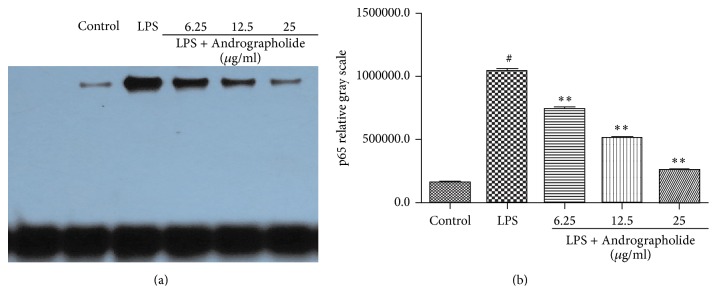
Effect of Andrographolide on nuclear level of transcription factor NF-kB. # indicates the control group compared with the LPS group (*P* < 0.05); *∗∗* indicates the Andrographolide group compared with the LPS group (*P* < 0.01). Results of the nuclear level of NF-kB probe binding activity (a) and gray scale (b) are representative of three (a-b) independent experiments.

We evaluated the effect of Andrographolide on the NF-*κ*B probe binding activity in NF-*κ*B pathway.  Figure 5 showed that the nuclear level of NF-kB probe binding activity in LPS group was significantly higher than that in the control group (*P* < 0.01). However, the nuclear level of NF-kB probe binding activity is significantly decreased in the nuclear extract obtained from Andrographolide treated cells and was significantly lower than that of the LPS group (*P* < 0.01).

### 3.5. Effect of Andrographolide on the Suppression of LPS-Induced NF-*κ*B/MAPK Pathway

**Figure 6:**
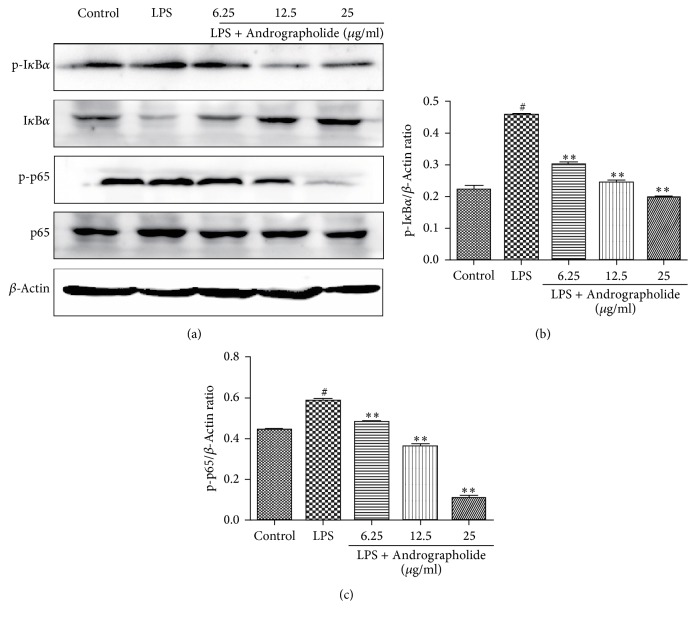
Effect of Andrographolide on the activation of NF-*κ*B pathways of RAW264.7 cells induced by LPS. # indicates the control group compared with the LPS group (*P* < 0.05); *∗∗* indicates the Andrographolide group compared with the LPS group (*P* < 0.01). Results of western blot (a), gray scale (b) and (c) are representative of three (a–c) independent experiments.

Since IkBa is phosphorylated after stimulation by LPS, followed by the release of the NF-*κ*B-p65 protein into the nucleus, and promotes the production of proinflammatory cytokines, we next evaluated the effect of Andrographolide on the inhibition of LPS-induced NF-*κ*B and MAPK pathways by a western blot. As shown in Figure 6, the levels of phosphorylated p65 and phosphorylated IkBa in the NF-*κ*B signaling pathway were significantly increased, while the levels of total IkBa in the NF-*κ*B signaling pathway were significantly reduced in LPS group. However, the levels of p65 and IkBa phosphorylation in the NF-*κ*B signaling pathway were significantly reduced in the Andrographolide groups (doses of 6.25, 12.5, and 25 g/ml) compared with the LPS group in a dose-dependent manner. In contrast, the level of total IkBa increased significantly with the increasing doses of Andrographolide. The above results indicate that the Andrographolide can suppress the LPS-induced NF-*κ*B pathway.

**Figure 7:**
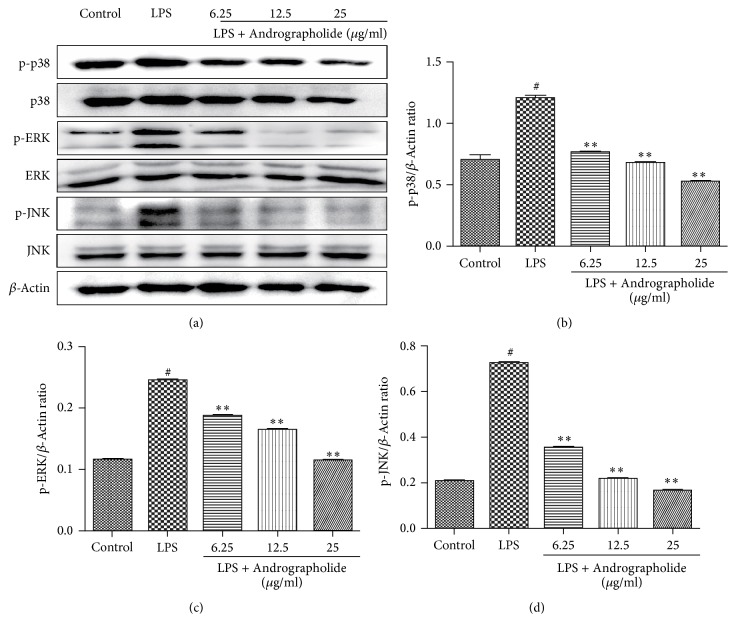
Effect of Andrographolide on the activation of MAPK pathways of RAW264.7 cells induced by LPS. # indicates the control group compared with the LPS group (*P* < 0.05); *∗∗* indicates the Andrographolide group compared with the LPS group (*P* < 0.01). Results of western blot (a), gray scale (b), (c) and (d) are representative of four (a–d) independent experiments.

As shown in Figure 7, the phosphorylation of JNK, ERK1/2, and p38 in the MAPK signaling pathway was significantly increased in LPS-stimulated samples, but the levels of p-JNK, p-ERK1/2, and p-p38 significantly decreased in the Andrographolide group (doses of 6.25, 12.5, and 25 g/ml) in a dose-dependent manner compared with the LPS group. These results suggest that Andrographolide can also suppress the LPS-induced MAPK pathway.

## 4. Discussion

*Andrographis* has many active components that have anti-inflammatory properties. Current research has shown that various active ingredients of* Andrographis* affect immune function, interacting with the platelet activating factor receptor, improving the nitric oxide levels in the body, scavenging oxygen free radicals, and inhibiting various proinflammatory cytokines, thus exhibiting a potent anti-inflammatory effect [14].

Andrographolide, the major active constituent extracted from* Andrographis*, occupies 70% of the* Andrographis* extract and has been reported to have many pharmacological properties, (e.g., it has antibacterial, antiviral, and antiplatelet properties, stimulates cellular differentiation, protects the liver, is a cholagogue, has antitumor properties, and is immunoregulatory). In addition, previous studies have shown that Andrographolide can inhibit increases in capillary permeability caused by xylene and acetic acid in mice [15]. Moreover, it has obvious anti-inflammatory effect, such as in laryngitis, upper respiratory tract infection, and rheumatoid arthritis [16–22].

Inflammation is a complex process involving the interaction between an organism and pathogens, with the results of these complex interactions to induce macrophage activation [23]. In addition, activated macrophages can eliminate invading infectious microbes and trigger the release of inflammatory cytokines, such as TNF-*α*, IL-1*β*, and IL-6, and then complete a variety of immune function in response to these cytokines [24]. Existing research shows that LPS is a potent inducer of inflammation and can stimulate macrophages to produce TNF-*α*, IL-1*β*, and IL-6. Thus, the organic damage caused by inflammation can be reduced by inhibiting the excessive production of inflammatory cytokines [25].

In the present study, an in vitro model of inflammation was induced by LPS in mouse RAW264.7 cells, followed by treatment with different concentrations of Andrographolide. Our results demonstrated that Andrographolide significantly inhibits the expression of TNF-*α*, IL-6, and IL-1*β* in LPS-stimulated RAW264.7 cells. Furthermore, we also found that Andrographolide has in vitro anti-inflammatory effects.

Andrographolide can inhibit the expression of TNF-*α*, IL-1*β*, and IL-6 in LPS-stimulated macrophages. Thus, the exploration of the anti-inflammatory mechanism of Andrographolide is significance [26]. The NF-*κ*B and MAPK signaling pathways are two extremely classical activation pathways in the process of LPS-induced signal transduction [27]. Under normal resting conditions, NF-*κ*B and IkBa aggregate into a trimer within the cytoplasm. In response to LPS, IkBa is predominately phosphorylated and subsequently degraded, which frees NF-*κ*B-p65 into the nucleus, and gene transcription of various proinflammatory factors is initiated [28, 29].

MAPK can also adjust the synthesis and release of the proinflammatory factors, p38, JNK, and ERK, which are three important pathways primarily involved in the inflammatory responses. When activated by external stimuli, p38, JNK, and ERK phosphorylation is increased and related proinflammatory cytokines will begin to be expressed [30]. Furthermore, the present study explores the anti-inflammatory mechanism of Andrographolide regarding the NF-*κ*B and MAPKs pathways. Our results demonstrated that LPS could induce the phosphorylation of IkBa, NF-*κ*B-p65, p38, JNK, and ERK, but the phosphorylation of IkBa, NF-*κ*B-p65, p38, JNK, and ERK was significantly inhibited when the cells were treated with Andrographolide. Consequently, the inhibition of the NF-*κ*B and MAPK pathways and the production of proinflammatory cytokines were inhibited, thus indicating that its anti-inflammatory effect was exerted via these pathways.

## 5. Conclusions

Andrographolide has anti-inflammatory effect and significantly inhibits the expression of TNF-*α*, IL-6, and IL-1*β* in LPS-stimulated RAW264.7 cells. Its anti-inflammatory mechanism may be through the inhibition of NF-*κ*B and MAPKs signaling pathway.

## Figures and Tables

**Table 1:** The TNF-*α*, IL-1*β*, and IL-6 primers.

Genes	Primer	Sequence 5′ → 3′	Product size (bp)
*TNF-α*	Sense	GTCTCAGCCTCTTCTCATTC	128
	Antisense	CATAGAACTGATGAGAGGGA	
*IL-1β*	Sense	AAATACCTGTGGCCTTGGGC	101
	Antisense	CTTGGGATCCACACTCTCCAG	
*IL-6*	Sense	GAGTCCTTCAGAGAGATACAG	125
	Antisense	CTGTGACTCCAGCTTATCTG	
*β-Actin*	Sense	CTTCATTGACCTCAACTACATGG	134
	Antisense	CTCGCTCCTGGAAGATGGTGAT	
